# VEGF (vascular endothelial growth factor) provides antimicrobial effects via autophagy and lysosomal empowerment in endothelial cells

**DOI:** 10.1080/27694127.2022.2137755

**Published:** 2022-10-24

**Authors:** Shiou-Ling Lu, Takeshi Noda

**Affiliations:** Center for Frontier Oral Sciences, Graduate School of Dentistry, Osaka University, Suita, Osaka, Japan

## Abstract

Xenophagy is an intracellular defense mechanism against invading pathogens. *Streptococcus pyogenes* (group A *Streptococcus*, GAS) is efficiently eliminated by this process in epithelial cells. However, we previously reported that the efficacy of xenophagy differs among cell types; intrinsic deficits in endothelial cell xenophagy failed to suppress GAS growth. Considering that the basal level of xenophagy is lower in endothelial cells as compared to epithelial cells, additional stimulation may be required to enhance this activity. Recently, we reported that VEGF (vascular endothelial growth factor) is the key factor facilitating xenophagy and lysosomal activity in endothelial cells. These processes further lead to the efficient killing of invading GAS. The cAMP-IP_3_-Ca^2+^ axis of the signaling pathway that activates TFEB (transcription factor EB) supports the transduction of the signal from VEGF. In addition, the severity of sepsis was observed to correlate with low VEGF concentrations in serum, both in a mouse model and in human patients. VEGF administration reduces mortality in a GAS sepsis model. Based on these findings, we propose that VEGF boosts the intracellular defense system of the endothelium by providing a strong blood vessel barrier to prevent bacterial dissemination.

*Streptococcus pyogenes*, also known as group A *Streptococcus* (GAS), is a pathogenic bacterium that causes a wide range of infectious diseases and is detrimental to human health. This bacterium is listed among the top ten pathogens that cause human bacterial infections, and serious invasive GAS infections have increased worldwide during the past two decades. Generally, GAS inhabits the skin and throat without any symptoms or causes only mild illnesses such as pharyngitis, impetigo, and scarlet fever. However, infection through skin injury in populations with immunocompromised conditions, such as among children or elderly people, causes life threatening disease, such as streptococcal toxic shock syndrome, sepsis, bacteremia, and necrotizing fasciitis, which results in GAS being labelled as “flesh-eating bacteria”. Furthermore, in some cases, cured patients develop non-bacterial sequelae, such as post-streptococcal glomerulonephritis and rheumatic heart disease.

GAS was initially regarded as an extracellular pathogen, but recently, it has become evident that some of them invade host cells via the endocytic pathway. Furthermore, some GAS escape the endocytic pathway and enter the cytosol by utilizing secreted pore-forming endotoxins such as streptolysin O. However, most of these GAS in the cytosol are eventually captured by host cell xenophagy, a type of macroautophagy/autophagy that selectively targets invading entities, resulting in lysosomal killing. One possible reason for the recent increase in severe cases is that certain GAS strains have evolved to be more invasive and have become capable of breaching deep into tissues or organs. Blood vessels normally serve as a robust physical barrier to such GAS invasion between the tissues and the blood circulation leading to sepsis; however, we reported in previous studies that the situation is more complex. Endothelial cells in blood vessels exhibit a higher GAS internalization efficacy than epithelial cells. Additionally, GAS invasion causes endothelial cell death. More significantly, blood vessel endothelial cells show low competency in xenophagy, which allows the intracellular proliferation of GAS. Considering the relatively nutrient-rich and germ-free environments of blood vessel endothelial cells in healthy, conditioned bodies, extensive autophagic activity may be considered as a low priority. An additional factor may be needed to sufficiently boost xenophagy in crucial disease conditions, such as sepsis.

Recently, we reported that VEGF has antimicrobial effects in endothelial cells in addition to their well-established pro-growth role in blood vessels [1]. Although GAS can proliferate inside endothelial cells after invasion, this proliferation is greatly suppressed by different commercial cell culture media used in previous studies. By comparing the ingredients of each medium, we determined that VEGF efficiently suppresses the intracellular growth of GAS in endothelial cells. VEGF can be supplied by the endothelial cells themselves and function in an autocrine manner at a level that is sufficient to support the basal growth of endothelial cells. However, this level is much lower than that required for the suppression of intracellular GAS growth. Therefore, augmentation of VEGF to increase VEGF signaling increases GAS clearance in endothelial cells.

VEGF promotes xenophagy by targeting intracellular GAS in endothelial cells. In VEGF-non-treated cells, LC3-positive GAS-containing structures are mostly single-membrane-bound LC3-associated phagocytosis-engaged phagosomes (LAPosomes) but not double-membrane-bound autophagosomes. However, in VEGF-treated cells, LC3-positive autophagosomal membrane structures surrounding GAS are frequently observed (Figure 1). Furthermore, VEGF-mediated GAS clearance is also observed in autophagy-deficient *atg7* KO endothelial cells, although this occurs to a lower extent. This latter clearance is due to the promotion of lysosomal and/or LAPosomal function by VEGF. Without sufficient VEGF, proper acidification in lysosomes and LAPosomes required for bacterial killing is not achieved in endothelial cells. These arguments are supported by the observed activation of TFEB, which upregulates the biogenesis of lysosomal and autophagy-related proteins. Knockdown of the TFEB pathway blocks VEGF-mediated antibiotic activity. This signal is transduced through the cAMP-IP_3_-Ca^2+^ axis downstream of VEGF (Figure 2).

**Figure 1.**
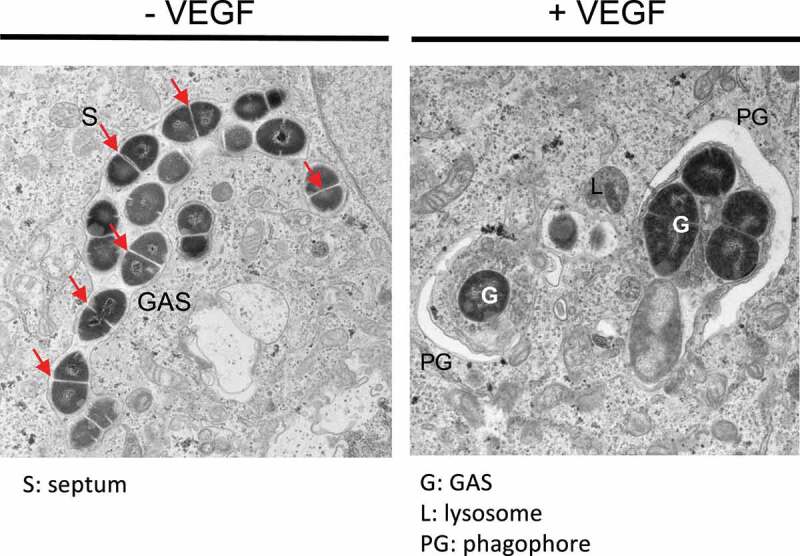
VEGF (vascular endothelial growth factor) facilitates autophagy targeting group A *Streptococcus* (GAS) in endothelial cells. Left image: In VEGF-untreated endothelial cells, GAS escapes into the cytosol and proliferates. The septum (S) is shown as a characteristic of division within the host cells. Right image: In VEGF-treated endothelial cells, a phagophore membrane (PG) surrounds the bacteria (G, GAS). Adopted and modified from Lu et al. (2022) https://doi.org/10.1128/mbio.01233-22 under the terms of the Creative Commons Attribution 4.0 International license.

**Figure 2.**
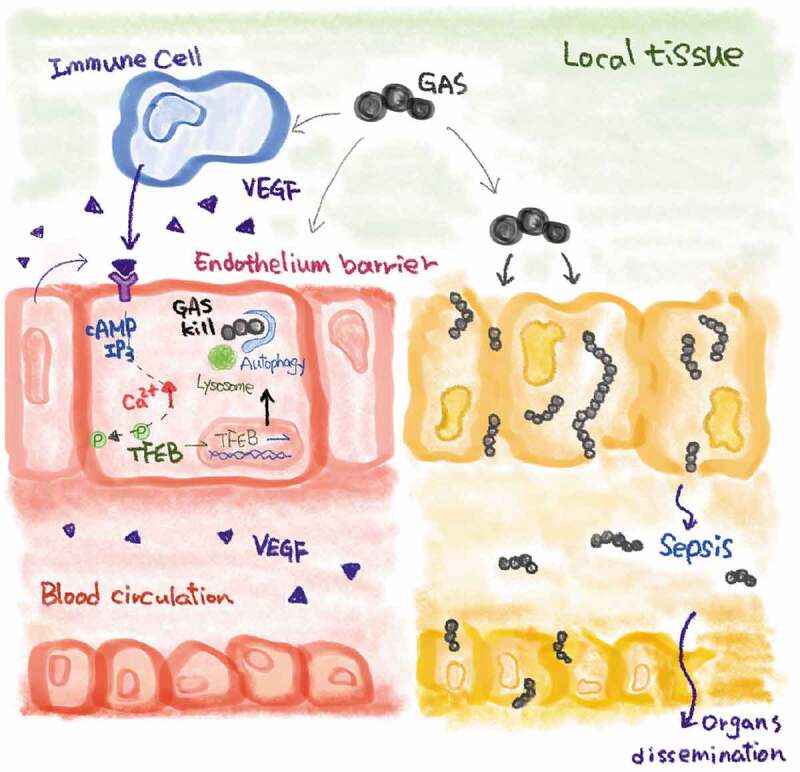
VEGF-mediated antimicrobial effects in endothelial cells.

In an *in vivo* GAS infection mouse model, serum VEGF levels are lower in mice with highly severe symptoms than in mice with milder symptoms. A similar correlation is observed in patients with GAS infections. Administration of VEGF restores the survival rate in the mouse model of GAS sepsis. VEGFs are produced by various immune cells that are mobilized to sites of inflammation. Based on these results, we propose a model in which during acute GAS infection, the local VEGF level increases at the site of infection, which then boosts intracellular autophagy and lysosomal competency in the endothelium to provide a more robust blood vessel barrier. Consequently, bacterial dissemination is suppressed. Insufficient establishment of barrier function due to the failure of this circuit may be correlated with severe symptoms. Promising vaccines against GAS are still under development, which allows the continued prevalence of GAS infectious diseases. Further research into this model is expected to lead to the development of efficient therapeutics for GAS infections with severe symptoms in the future.
